# *QuickStats:* Life Expectancy at Birth, by Sex —  United States, 2019–2022

**DOI:** 10.15585/mmwr.mm7313a5

**Published:** 2024-04-04

**Figure:**
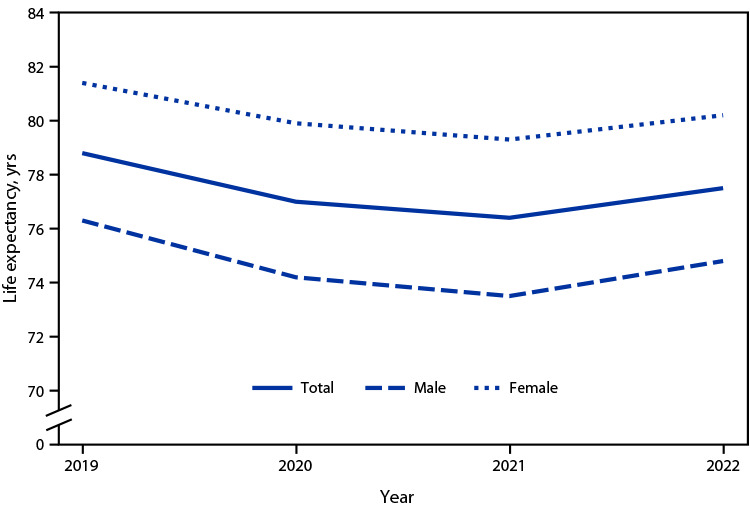
Life expectancy at birth for the U.S. population in 2022 was 77.5 years, an increase from 76.4 years in 2021. Although life expectancy rose in 2022 for the first time since the COVID-19 pandemic began, it remains lower compared with prepandemic life expectancy in 2019 (78.8 years). This pattern was similar for males and females.

**Supplementary Table: **https://stacks.cdc.gov/view/cdc/151563

**Sources:** National Vital Statistics System, United States Life Tables, 2021 (https://www.cdc.gov/nchs/data/nvsr/nvsr72/nvsr72-12.pdf); Mortality in the United States, 2022 (https://www.cdc.gov/nchs/products/databriefs/db492.htm).

